# Quantitative Expression of *SFN*, lncRNA CCDC18-AS1, and lncRNA LINC01343 in Human Breast Cancer as the Regulator Biomarkers in a Novel ceRNA Network: Based on Bioinformatics and Experimental Analyses

**DOI:** 10.1155/2022/6787791

**Published:** 2022-09-12

**Authors:** Mehrnoush Rishehri, Tahereh Etemadi, Leila Pisheh, Ghazaleh Koufigar, Mansoureh Azadeh

**Affiliations:** ^1^Zist Fanavari Novin Biotechnology Institute, Isfahan, Iran; ^2^Department of Medical and Pharmaceutical Science, University of Genova, Genova, Italy; ^3^Department of Biology, Rasht Branch, Islamic Azad University, Rasht, Iran

## Abstract

Breast cancer (BC) is one of the leading cancers in the world, which has become an increasing serious problem. In this context, reports demonstrate that some long noncoding RNAs (lncRNAs) play significant regulatory roles in breast tumorigenesis and BC progression via various pathways and act as endogenous RNAs. Finding their dysregulation in cancer and evaluating their interaction with other molecules, such as short noncoding RNAs “microRNA (miRNAs)” as well as various genes, are the most important parts in cancer diagnostics. In this study, after performing GSEA and microarray analysis on the GSE71053 dataset, a new ceRNA network of CCDC18-AS1, LINC01343, hsa-miR4462, and *SFN* in BC was detected by bioinformatics analysis. Therefore, the expression of *SFN*, CCDC18-AS1, and LINC01343 was quantitatively measured in 24 BC and normal paired tissues using qRT-PCR. CCDC18-AS1, LINC01343, and *SFN* were expressed higher in BC than in the control (normal paired) tissues based on qRT-PCR data. Furthermore, a significant positive correlation was observed between CCDC18-AS1 and LINC01343 expression in the samples investigated in this study. The investigation of clinicopathological parameters showed that *SFN* was highly expressed in tumor size of <5 cm and in nonmenopausal ages, while CCDC18-AS1 and LINC01343 indicated a high expression in stages II-III and III of BC, respectively. The overall survival analysis displayed high and low survival in patients with high expression of *SFN* and CCDC18-AS1, respectively. The ROC curve analysis disclosed that *SFN*, CCDC18-AS1, and LINC01343 might be suggested as potential biological markers in BC patients. The high expression of CCDC18-AS1, LINC01343, and *SFN* in BC samples suggests their potential role in BC tumorigenesis and could be considered hallmarks for the diagnosis and prognosis of BC, although this will require further clinical investigations.

## 1. Introduction

Breast cancer (BC) is a complex neoplastic disease with various stages, from benign to invasive malignant tumors, and represents the most commonly diagnosed cancer in women [1]. Despite remarkable advances in diagnosis and treatment in recent years, the complexity of the molecular pathways underlying BC has largely prevented the development of targeted treatments for this disease. In recent decades, the role of noncoding RNAs in gene regulation has attracted widespread attention in medical research [2]. Long noncoding RNAs (lncRNAs) and miRNAs are presented as two major classes of noncoding RNAs whose functions in a variety of cancers such as BC have been extensively studied; therefore, some lncRNAs/miRNAs are regarded as promising therapeutic targets in BC [3].

LncRNAs with a length greater than 200 nucleotides are defined as a major class of non-protein-coding RNAs and are involved in many physiological and pathological processes [4]. It is well established that lncRNAs are involved in various steps of cancer pathophysiology, including proliferation, angiogenesis, immortality, invasion, and metastasis [5]. MiRNAs are short noncoding RNAs, typically 17–25 nucleotides in length, and their dysregulation is associated with an increased risk of cancers, including BC [6].

LncRNAs and miRNAs not only control gene transcription but also participate in gene expression through the lncRNA-miRNA-mRNA network. It is noteworthy that lncRNAs act as competing endogenous RNAs (ceRNA) and can function against miRNAs to regulate the expression of neighboring genes in the physiological process, influencing tumor development [7, 8]. The ceRNA theory assumes that lncRNAs and mRNAs sharing the same miRNA response elements (MREs) compete for linkage to the same miRNA, thereby regulating each other's expression [9]. The performance of the lncRNAs-associated ceRNA network has been demonstrated in several cancers, namely, breast, gastric, glioblastoma [4, 10, 11]. In addition, dysregulation of lncRNA can also intervene in favor of hematological malignancies, e.g., leukemia [12]. LncRNAs also play an important role in several human diseases such as diabetes [13], infertility, e.g., lncRNA H19 [14], as well as cardiovascular diseases [15]. The interaction of lncRNAs and miRNAs creates a complex regulatory network through which it ultimately modulates gene and/or protein expression at many levels, i.e., transcriptional, posttranscriptional, and posttranslational [16].

The *SFN* gene (14-3-3*σ* proteins or stratifin) is one of various genes implicated in several cancer pathways, as it plays multifunctional regulatory role in several cellular processes related to cancer pathophysiology, including cell cycle progression, cell growth, and apoptosis [17]. The functions of SFN may vary depending on the organs and/or tissues, and several studies have shown that upregulation of SFN promotes cancer of pancreas [18], head and neck [19], lower gastrointestinal tract [20], lung [21], and gallbladder [22]. Nonetheless, SFN has been identified as well as a tumor suppressor/modulator gene in colon [23], ovaries [24], breast [25], bladder [26], and lung [27] and its expression was downregulated in these cancers [18–22]. In most cancers, including BC, this downregulation is known to be due to SFN gene inactivation, generally through promoter methylation; for this reason, DNA hypermethylation in the promoter of SFN has been used as a biomarker for cancer diagnosis [28].

In this study, we used gene set enrichment analysis (GSEA) and microarray analysis to identify the most differentially expressed genes (DEGs) in BC and control breast tissues from the GEO database. Finally, the *SFN* gene was selected as a target for further investigation. For data validation, the expression of the *SFN* gene, the LINC01343, and CCDC18-AS1 lncRNAs (which were determined by bioinformatic analysis) was analyzed by qRT-PCR in tumor and paired control breast tissues.

## 2. Materials and Methods

### 2.1. Identification of Differentially Expressed mRNA Using GSEA

GSEA (Gene Set Enrichment Analysis) represents a proper computational method for identifying genes with a common biological function and pathway enrichment. In this study, GSEA was performed using the GSEA V 4.1.0 software to analyze the GSE71053 dataset [29–31] extracted from Gene Expression Omnibus (GEO) (https://www.ncbi.nlm.nih.gov/geo/). Subsequently, the Wilcoxon rank-sum test was used to analyze differentially expressed genes (DEGs). False discovery rate (FDR) <0.25 was considered as the significance threshold.

### 2.2. Identification of Differentially Expressed mRNA Using Microarray Analysis

We used the GSE71053 entry from the GEO database (https://www.ncbi.nlm.nih.gov/geo/) and analyzed it to find the DEGs in BC samples compared to normal samples. Data were analyzed using the “limma” [32] and “affy” [33] packages in R software 4.1.0, and graphs and figures of the microarray analysis were drawn by ggplot2 and pheatmap packages. Based on the distribution of expression data of all genes investigated in this experiment, genes with logFC greater than the third quartile were selected as overexpression genes (logFC = 0.156276), and those with logFC less than the first quarter were selected as low-expressed genes (logFC = −0.194726).

### 2.3. Bioinformatic Analyses

The ceRNA network was created based on the hypothesis that lncRNAs affect miRNAs to regulate the mRNA's activity and expression. lncRNA-miRNA-mRNA network was established using three databases: (1) miRWalk V.3 (https://mirwalk.umm.uni-heidelberg.de/) [34], (2) LncRRIsearch-rtools V.1.00 (https://rtools.cbrc.jp/LncRRIsearch/n.d) [35], and (3) lncBase Module-DIANA TOOLS V.2 (https://diana.imis.athena-innovation.gr/DianaTools/n.d) [36]. Then, the regulatory network was built based on lncRNAs-miRNA-mRNA interaction pairs and visualized by PowerPoint (Microsoft, 2019) software.

### 2.4. Patients and Tissue Samples

In this study, samples were collected from 24 paired tumors and adjacent nontumor tissues after surgical resection (mean of the ages: 47 ± 12.59). After washing with distilled water, the tissues were immediately immersed in RNA-later (Yekta Tajhiz Azma, Iran). They were stored at −70°C before usage. None of the patients had previously received radiotherapy or chemotherapy. The pathological characteristics of patients were reviewed by the pathologist and summarized in Table 1. All patients signed written informed consents before the beginning of the study. In addition, study protocols in this experiment were approved by the Ethics Committee from Al-Zahra Hospital Isfahan University of Medical Sciences, based on the Helsinki Declaration of 1964.

### 2.5. Total RNA Extraction and Real-Time Quantitative Reverse Transcription PCR

The total RNA was extracted from tumor and normal tissues samples using YTzol Kit (YTzol Pure RNA, Yekta Tajhiz Azma, Iran). According to the manufacturer's protocol, cDNA was synthesized using the cDNA Synthesis Kit (Takara, Japan). Specific PCR primers were designed using Oligo7 for the SFN gene, CCDC18-AS1, LINC01343, and GAPDH (listed in Table 2). Quantitative RT-PCR assay was performed using a real-time PCR instrument (Biomolecular Systems, Magnetic Induction Cycler (MIC), Australia). GAPDH was used as the housekeeping gene, and SFN CT (the cycle threshold) values were normalized with the CT value of GAPDH [37, 38].

### 2.6. Statistical Analysis

QRT-PCR data was analyzed using -ddCT method. Statistical analysis was performed using paired *t*-test and Wilcoxon test to compare and analyze expression data between BC and normal tissue samples using GraphPad Prism software version 8.0 (GraphPad Software, San Diego) and GenEx software (version 6). DEGs analysis was assessed using Bioconductor packages in RStudio software (version 4.0.2). GraphPad Prism also created receiver operating characteristic (ROC) curves. In addition, the Kruskal-Wallis test was performed for clinicopathological analysis. Spearman correlation test was performed to detect the correlation between lncRNAs and genes in BC. Survival curves were plotted using GEPIA2 [39]. In GSEA, FDR <0.25 and *p* ≤ 0.05 were considered statistically significant in all other analyses [38, 40].

## 3. Result

### 3.1. Gene Set Enrichment Analysis (GSEA) in Response to BC

**Figure 1:**
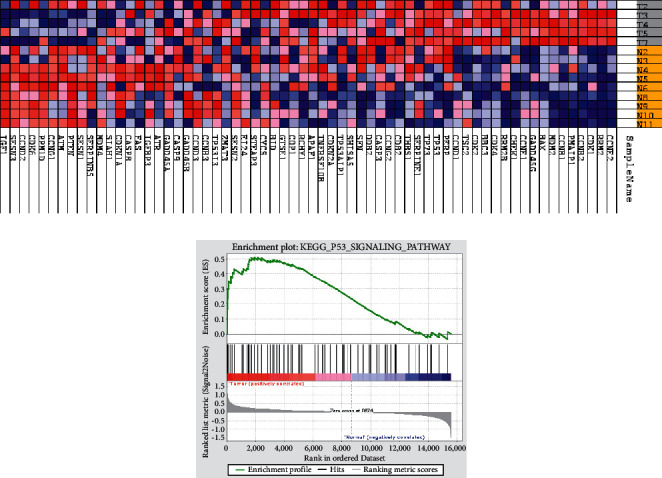
Figures display heatmaps and enrichment plots from GSEA. (a) Heatmap shows the name of groups in a row and the name of genes in a column. The red color in the heatmap and enrichment plot indicates a strong association between gene expression levels and the phenotype of BC. In contrast, the blue color shows a negative correlation between the level of gene expression and the phenotype of the normal breast. (b) The results of enrichment by GSEA show the major signaling pathways linked to the selected gene.

To extract biological information, GSEA was used to analyze gene expression data. In this study, the upregulated genes were annotated from 34846 genes, which were extracted from the GSE71053 dataset and are available in Figure 1(a). Heatmap for DEGs was created by comparison of the high-score and low-score groups in BC. GSEA was performed against Kyoto Encyclopedia of Genes and Genomes (KEGG) or hallmark gene set signatures to get further information at the gene set level and the main implicated pathways (Figure 1(b)). According to the heatmap, GSEA presented that *SFN* is the highly enriched gene in BC samples (FDR <0.25). Besides, the P53 pathway was significantly enriched using GSEA of KEGG v99.0 (Figure 1(b)). Our findings demonstrate the important use of GSEA for gene expression analysis and highlight novel cancer cell signaling data.

### 3.2. Microarray Analysis

**Figure 2:**
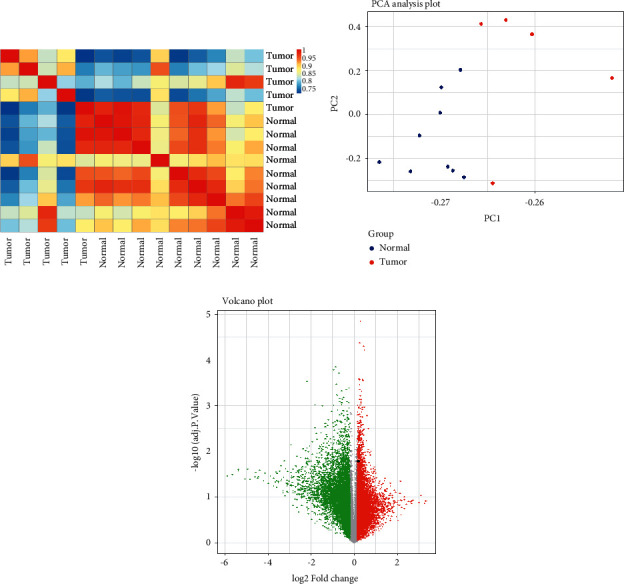
Analysis of DEGs in dataset GSE71053 using R software (a, b, c). (a) The heatmap figure represents the correlation between the two groups normal and BC in 54675 DEGs; (b) principal component analysis (PCA) between healthy and BC tissues based on the GSE71053 dataset. (c) Volcano plot of DEGs in the GSE71053 dataset. SFN is showed by a black dot in the graph. *X*-axis represents log2 fold change (FC) and *Y*-axis represents −log10 (adjusted *p* value). Red dots depict the low expression of genes (*n* = 8711), and green dots indicate the high expression of genes (*n* = 8712) in the GSE71053 database.

R software was used for the preparation, normalization, and utilization of the GSE71053 dataset. According to the results of the differentially expressed genes (DEGs) analysis using the “limma” and “affy” packages, *SFN* was selected as a significant differential expression gene (logFC = 0.8226, *p* = 0.02568) between upregulated DEGs in BC (Figures 2(a) and 2(c)). The quality of microarray samples was evaluated by principal component analysis (PCA) (Figure 2(b)).

### 3.3. Construction of the ceRNA Regulatory Network in BC

**Figure 3:**
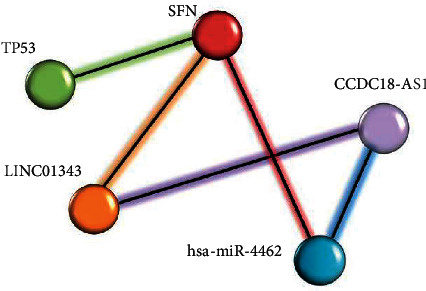
The novel ceRNA interaction (lncRNAs-miRNA-mRNA) associated with tumor networks of BC shows that hsa-miR-4462 and LINC01343 targeted SFN. miRNA and LINC01343 both have interaction with CCDC18-AS1.

In this study, we used miRWalk V.2 [34], LncRRIsearch-rtools V.1.00 [35], and DIANA-LncBase V.2 (DIANA Tools) [36] to construct a regulatory network in BC. First, we predicted the miRNAs that interacted with SFN (acquired from microarray and GSEA) using miRWalk V.2, and it was set based on a “0.95” score, “5UTR,” and “RNAhybrid.” Secondly, we used LncRRIsearch-rtools V.1.00 to assess the lncRNAs-mRNA interactions related to our mRNA, and finally, DIANA-LncBase Version 2 identified the lncRNA-miRNA interactions with 0.956 and 0.911 scores. Ultimately, we concluded the lncRNAs, miRNA, and mRNA interactions and created a lncRNA-related ceRNA network. In addition, using this method, the TP53 protein has been shown to coexpress (and thus interact) with the SFN protein (Figure 3) using String web V.11.5 (https://string-db.org/) [41].

### 3.4. Expression Levels of *SFN*, lncRNAs CCDC18-AS1, and LINC01343 in BC

**Figure 4:**
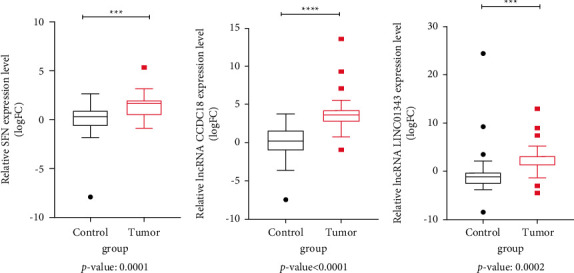
The expression level of *SFN*, CCDC18-AS1, and LINC01343 in tumor tissues (*n* = 24) compared to the normal tissues (*n* = 24) by qRT-PCR. Figures demonstrated the high expression of *SFN*, CCDC18-AS1, and LINC01343 in tumor samples compared to the normal samples (● outlying data of control groups, ■ outlying data of tumor groups, ^*∗∗∗*^*p* value <0.001,^*∗∗∗∗*^*p* value <0.0001).

The expression of the *SFN* gene and two lncRNAs, including CCDC18-AS1 and LINC01343, was acquired based on bioinformatic as well as microarray analysis and assessed in tissue samples of BC (*n* = 24) and control (*n* = 24) using the qRT-PCR method. SFN gene expression in cancer tissues was significantly increased (*p*=0.0001) in comparison to control tissues (Figure 4(a)). In addition, a significant elevation of CCDC18-AS1 and LINC01343 expression was observed in the cancer tissues as compared to control groups (*p* < 0.0001 and *p*=0.0002, respectively) (Figures 4(b) and 4(c)). In other words, we identified higher expression of 2.366-fold of *SFN* (|LogFC| = 1.243), 12.915-fold of LINC01343 (|logFC| = 3.691), and 10.584-fold of CCDC18-AS1 (|logFC| = 3.397) in the cancer tissue in comparison to the control group.

### 3.5. Diagnostic Performance of Studied mRNA and lncRNAs for BC Detection

**Figure 5:**
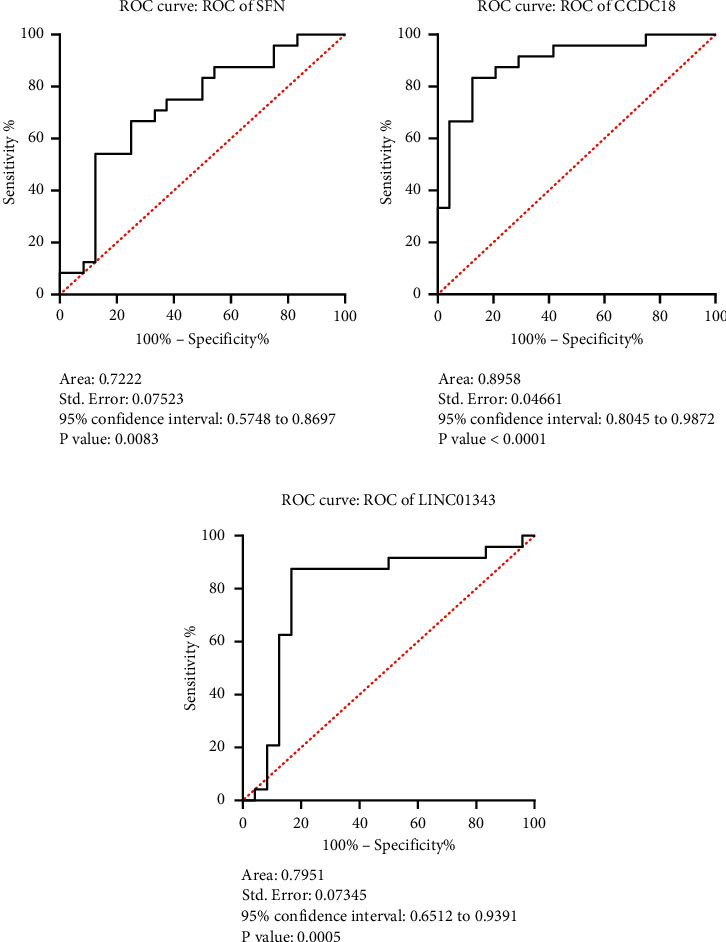
The ROC curve of sensitivity versus specificity of *SFN*, LINC01343, and CCDC18-AS1 in BC based on their expression resulting from qRT-PCR was constructed by GraphPad. In these plots, an excellent model with AUC near 1 has a good measure of separability.

To assess the potential of lncRNAs and mRNA as diagnostic biomarkers for assessing the health or disease status, these lncRNAs and mRNA were analyzed using RT-PCR by the receiver operating characteristic (ROC) analysis in both groups. The ROC curve analysis revealed *SFN* gene as a potential biomarker (AUC: 0.7222, *p*=0.0083), the LINC01343 gene as a good biomarker (AUC: 0.7951, *p*=0.0005) in discriminating BC patients from healthy individuals, and the CCDC18-AS1 gene as the strongest biomarker (AUC: 0.8958, *p* < 0.0001) (Figures 5(a)–5(c)). These findings highlight each of the three genes as a promising screening tool in different respects.

### 3.6. lncRNAs Expression Level is Positively Correlated in BC

**Figure 6:**
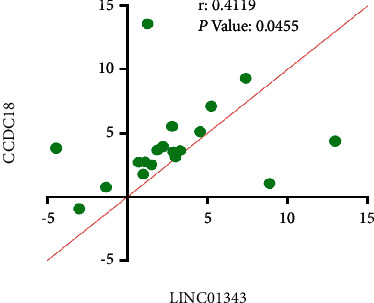
According to the spearman correlation analysis, there was a significant positive correlation between the expression level of CCDC18-AS1 and LINC01343 in BC samples.

In order to explore the relationship between SFN, LINC01343, and CCDC18-AS1, the correlation between each of the three mRNAs in BC was investigated. It was found that there is a significant (positive) correlation between LINC01343 expression levels and CCDC18-AS1 (*r* = −0.4119, *p*=0.0455). This implies that the higher expression of CCDC18-AS1 lncRNA was linked with the increased expression of LINC01343 in BC patients (Figure 6).

### 3.7. Clinicopathological Analyses in BC Sample Tissues

**Figure 7:**
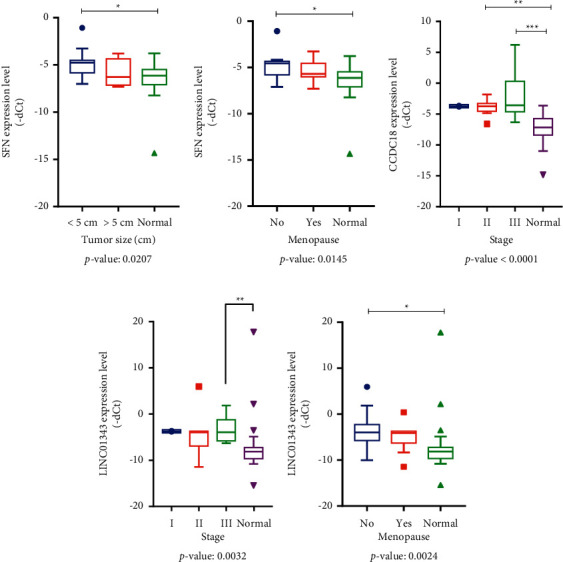
Clinicopathological analysis of BC tissues. QRT-PCR data of *SFN*, LINC01343, and CCDC18-AS1 were analyzed according to the clinicopathological parameters (stage, tumor size, and menopause ages) by KruskalWallis test.

We further investigated whether the expression levels of SFN, LINC01343, and CCDC18-AS1 are statistically related to the clinical features of BC. Our findings are that the expression of *SFN* gene was significantly higher in the BC tissues with tumor size less than 5 cm and also in nonmenopause ages compared to the normal group (*p*=0.0207 and *p*=0.0145, respectively) (Figures 7(a) and 7(b)). As shown in Figures 7(c) and 7(d), no significant difference in the expression of lncRNAs was observed in early stage of BC when comparing normal and tumor tissues, while the expression of the CCDC18-AS1 was significantly increased in stages II-III of disease compared to the control group (|−dct| = 1.5 and 2, respectively, and *p* < 0.0001). On the other hand, LINC01343 also showed significant overexpression at stage III of BC (|−dct| = 4 and *p*=0.0032), as well as nonmenopause ages (|−dct| = 4 and *p*=0.0024) compared to the control group (Figure 7(d) and 7(e)).

### 3.8. Survival Analysis

**Figure 8:**
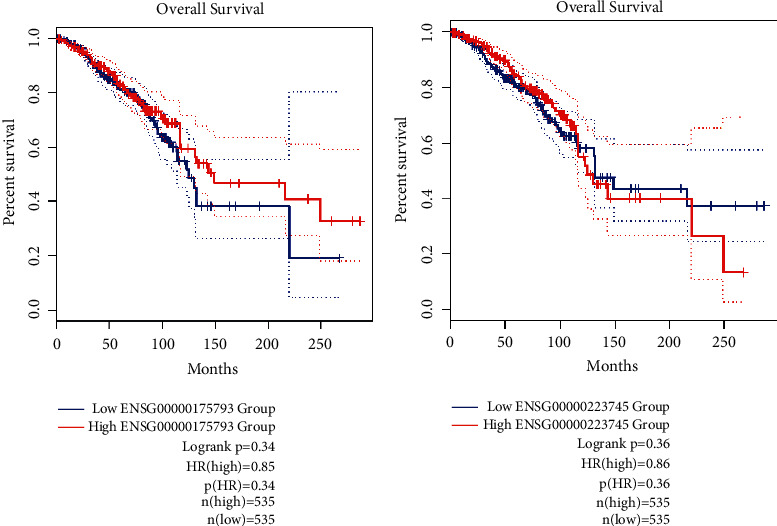
The overall survival (OS) analyses based on the cancer type in BC using GEPIA2 data. (a) *SFN*, (b) CCDC18-AS1.

GEPIA (Gene Expression Profiling Interactive Analysis) web server provides publicly available, customized analysis for gene expression analysis based on TCGA, GTEx, and RNA-seq databases from tumor and normal samples (https://gepia.cancer-pku.cn/) [39]. Using this database, we performed differential expression analysis related to the *SFN* gene and CCDC18-AS1 for patients with BC. In the expression analysis, the overall survival anticipated that patients who had positive *SFN* expression survived significantly more than patients who had negative *SFN* expression (*p* value = 0.34, Figure 8(a)). Furthermore, patients in the CCDC18-AS1 negative group had better survival than patients with positive CCDC18-AS1 expression (*p* value = 0.36, Figure 8(b)).

## 4. Discussion

BC remains a major threat to the health of women in all ages and is the second leading cause of malignant death worldwide [42]. Therefore, identifying the molecular factors and mechanisms underlying the development and progression of BC may be effective in early diagnosis and (timely) targeted treatment of these patients.

A large number of studies highlighted that the interactions between lncRNAs, miRNAs, and mRNAs constitute specific regulatory networks and influence genes expression in cancer [43, 44]. In this study, by using several bioinformatics tools, we predicted the lncRNA-miRNA-mRNA regulatory network and proposed a novel ceRNA regulatory network (CCDC18-AS1, LINC01343, hsa-miR-4462, *SFN*) underlying BC pathophysiology. Traditionally, lncRNAs have been considered as a “sponge” or competitive endogenous RNA (ceRNA), interacting with miRNA, and decrease the inhibition effect of miRNAs on mRNAs [45]. Therefore, this study suggests that CCDC18-AS1 can control *SFN* gene expression by repressing hsa-miR-4462, whereas LINC01343 can directly targets the *SFN* gene [35].

Following the identification of *SFN* as a potentially upregulated gene in BC (based on GSEA and microarray analysis) we also showed a potential interaction of *SFN* with CCDC18-AS1 and LINC01343 based on *in silico* protein-protein interaction. Our experimental analysis also revealed high expression of *SFN*, CCDC18-AS1, and LINC01343, supporting the oncogenic activities of these factors in the context of BC pathophysiology. Last but not least, our analyses indicated the high expression of CCDC18-AS1 and LINC01343 in BC at stages II-III and III, suggesting that the dysregulation of these factors may play a role implicated in both tumor invasion and metastasis. In contrast, high expression of *SFN* in tumors of size <5 cm can present its oncogenic effect in very early stages of BC, such as tumor initiation.

A study of colorectal carcinoma (CRC) has reported a negative correlation between CCDC18-AS1 and downregulated BGs (bait genes), suggesting that CCDC18-AS1 may be a potential oncogenic lncRNA associated with CRC [9]. Moreover, our results also showed that CCDC18-AS1 could be a potential biomarker in BC and that patients with negative expression of CCDC18-AS1 have a better survival compared to patients with positive expression of CCDC18-AS1.

LINC01343 has been shown to be associated with type 2 diabetes (T2D) and coronary artery disease (CARD) [46]. Consistent with the bioinformatics data, the upregulation of LINC01343 detected by qRT-PCR in our study may indicate its possible role in BC pathophysiology. In addition, our findings presented LINC01343 as a promising biomarker in BC which may be a hallmark for diagnosis and treatment of patients in the future.

It has been shown that lncRNAs exert their role as tumor suppressor or oncogene through interaction with miRNAs and mRNAs [9, 47]. In our study, CCDC18-AS1 and LINC01343 affect *SFN* gene and regulate its expression and function. *SFN* is one of the members of the 14-3-3 family, which is known as human mammary epithelial cell marker (HME-1) and has been directly associated with cancer. *SFN* has been reported to function as a tumor suppressor gene whose functional inactivation may be associated with tumorigenesis. Several studies support this hypothesis and have demonstrated downregulation of the *SFN* gene in several human cancers, including breast [48], ovaries [49], lung [50], liver [51], prostate [52], and oral cavity [53]. However, many conflicting studies have represented upregulation of *SFN* in the head and neck [14], gastric [54], pancreas [55], and colorectal cancers [20]. In our study, the *SFN* gene was also abnormally overexpressed in BC samples, defining *SFN* as an oncogene and a biomarker in BC. Similar to our results, studies have also reported that overexpression of *SFN* can be used as a better prognostic biomarker in gallbladder cancer [22] and the stroma of pancreatic ductal adenocarcinoma (PDAC) treatment [56]. In patients with esophageal squamous cell carcinoma (ESCC), a low level of *SFN* is associated with a poorer prognosis and survival rate [57]. Our analysis also showed the shorter overall survival in patients with low expression of *SFN* in BC. Furthermore, *SFN* could be positively implicated as an oncogene in a variety of diseases, including Alzheimer's disease, Parkinsonian syndromes, and autoimmune disorders affecting the central nervous system [58].

Considering the direct contribution of *SFN* to the cancer development, it seems that several molecular landscapes may control its downstream expression. P53, an important tumor suppressor gene, may be among the most important regulators of *SFN* expression [59]. Activation of p53 in response to cellular DNA damage and its direct binding to the promoter region leads to transactivation of *SFN* expression. Subsequently, *SFN* regulates the cell cycle by triggering G1/S mediated by p21. *SFN* specifically affects Cdk1/cyclin B1, cyclin-dependent kinase-2, and cyclin-dependent kinase-4 (CDK2, CDK4) and prevents cells from entering mitosis [28]. *SFN* negatively regulates the cell cycle and suppresses tumor. Overall, *SFN* exerts the tumor suppressor effects arresting the cell cycle by two different mechanisms: (a) by inhibiting the formation of the Cdc2-cyclin B1 complex (via the G2/M checkpoint) or (b) by blocking MDM2 (preventing MDM2-mediated ubiquitination of p53) [60].

On the other hand, it was reported that *SFN* could have positive effects on the cell cycle and also accelerate cell proliferation. Zhang et al. illustrated that *SFN* is a positive mediator of IGF-I receptor-induced cell proliferation. *SFN* interacts with the PI3K/Akt pathway independently of p53 and promotes the cell cycle progression in MCF-7 BC cells. Moreover, upregulation of the *SFN* gene was associated with an increase in cyclin D1 level in MCF-7 cells, shortening the duration of G1 phase, and accelerating the cell cycle [61]. Furthermore, SFN could positively contribute to the activation of receptor tyrosine kinases (RTKs) and prevent its degradation [62], leading to increase in the activation of the RTKs signaling pathway and induction of different cellular processes such as proliferation, growth, motility, and differentiation [63]. Accordingly, increased *SFN* levels appear to be associated with upregulation of p-Akt and cyclin D1, leading to the cell cycle progression [62]. In addition, elevated p-Akt level in response to the *SFN* can also be involved in tumorigenesis through activation of MDM2 and inhibition of p53 [64]. Furthermore, silencing of *SFN* is associated with the upregulation of proapoptotic proteins (Bim and Bax) in cholangiocarcinoma cells, suggesting the oncogenic potential of *SFN* through inhibition of apoptosis [62]. Moreover, the antiapoptotic function of *SFN* may be associated with its inhibitory interaction with Bad and Bax (both proapoptotic proteins) [65].

**Figure 9:**
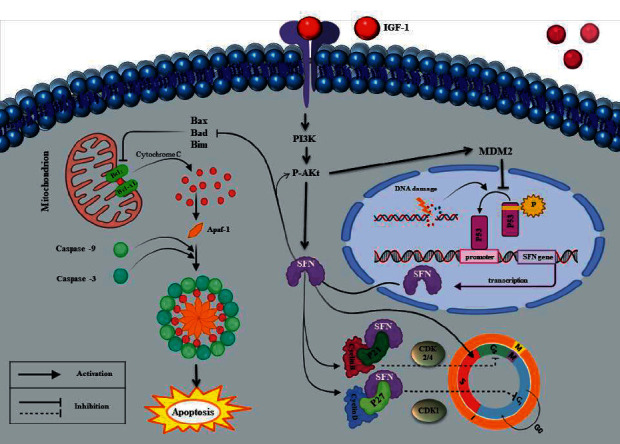
Possible mechanisms of *SFN* role in tumorigenesis. Although *SFN* dependent on P53 induces the cell cycle arrest, it can prevent apoptosis by sequestrating Bad and Bax, which symbolizes an excellent capability of *SFN* as an oncogene [65]. In addition, it may have roles in proliferation, growth, motility, differentiation, and metabolism through PI3K/Akt pathway [61].

As we have shown in Figure 9, upstream signaling pathways of IGF1 and RTKs can increase *SFN* level and activity in cancer cells [61, 62]. *SFN* can sequester phosphorylated Bad in the cytosol, thus preventing apoptosis exerted by Bad [65]. In addition, *SFN* can increase the cyclin D1 level and accelerate the cell cycle and proliferation [61]. Consequently, the accumulation of *SFN* can be proposed as a hallmark of BC. However, the direct role of *SFN* in inhibiting apoptosis and the cell cycle progression was not investigated in our study.

To better understand this ceRNA network, a significant correlation between CCDC18-AS1 and LINC01343 lncRNAs was observed experimentally, which could bioinformatically establish the interaction of these lncRNAs with *SFN*, which may induce the oncogene effect of *SFN* gene in the p53 pathway in the BC process. In this experiment, there were some drawbacks, such as limited access to human clinical samples and investigation of high-throughput genes within the study, and some of our graphs were obtained from the GEPIA2 online database, such as patients' survival. For future studies, we recommend that the expression level of *SFN*, CCDC18-AS1, and LINC01343 be examined in animal samples to determine the accurate expression pattern of these lncRNAs and *SFN* gene in BC.

## 5. Conclusion

Our study reported overexpression of *SFN*, CCDC18-AS1, and LINC01343 in BC tissues, which could describe them as novel and promising biomarkers for BC diagnosis. These findings could lead to a new understanding of the clinical significance of lncRNA-mediated ceRNA networks as potential prognostic biomarkers and therapeutic targets of BC. Nevertheless, these results need to be validated and confirmed by using a range of detailed statistical approaches in larger sample cohorts. According to the various downstream partner interaction changes, *SFN* does not seem to perform its crucial operations, in particular antiapoptotic action and maintenance of the G2 checkpoint. The identification of alternative molecular pathways in which *SFN*, CCDC18-AS1, and LINC01343 contribute to timely diagnosis of BC which may provide novel approaches for the prognosis and treatment of BC. Therefore, for future studies, investigating the exact molecular pathways of *SFN*, CCDC18-AS1, and LINC01343 in cancer may shed new light on the important and emerging role and function of these factors in tumorigenesis and lead to newer approaches for both the diagnosis and treatment of BC. Due to the limited data available on CCDC18-AS1 and LINC01343, functional studies on these lncRNAs will help to unravel their role at the cellular level in both health and diseases, which could also provide potentially useful information for BC diagnosis and treatment.

## Figures and Tables

**Table 1:** The pathological characteristics of patients.

Characteristics	Status	Number of patients
Stage	I	1 (4.2%)
	II	9 (37.6%)
	III	10 (41.6%)
	IV	0
	Unknown	4 (16.6%)

Age	<45	11 (45.8%)
	>45	13 (54.2%)
	Unknown	0 (0%)

Tumor size (TS)	<5 cm	14 (58.4%)
	>5 cm	6 (25%)
	Unknown	4 (16.6%)

Menopausal status	Yes	11 (45.8%)
	No	12 (50%)
	Unknown	1 (4.2%)

Lymph node	Yes	17 (70.8%)
	No	3 (12.6%)
	Unknown	4 (16.6%)

ER receptor	Positive	10 (41.6%)
	Negative	6 (25%)
	Unknown	8 (33.4%)

PR receptor	Positive	8 (33.4%)
	Negative	8 (33.4%)
	Unknown	8 (33.4%)

HER2/neu receptor	Positive	8 (33.4%)
	Negative	8 (33.4%)
	Unknown	8 (33.4%)

**Table 2:** Primers for real-time PCR analysis.

Gene name	Sequence primer PCR	
CCDC18-AS1	Reverse	5′-CAGCGTAAGGGTGGAACAG-3′
	Forward	5′-AAACTGTCGTCCTGGTGGG-3′

LINC01343	Reverse	5′-GTCACCAGCTCATTCACGC-3′
	Forward	5′-ATCTGTCTTAGATTGGGGGTC-3′

*SFN*	Reverse	5′-AGCCCTTTGGAGCAAGAACAG-3′
	Forward	5′-ACAACCTGACACTGTGGACG-3′

*GAPDH*	Reverse	5′-ACAACCTGACACTGTGGACG-3′
	Forward	5′-ACAACCTGACACTGTGGACG-3′

## Data Availability

The data used to support the findings of this study are available from the corresponding author upon request.

## References

[1] Hong D., Fritz A. J., Zaidi S. K. (2018). Epithelial-to-mesenchymal transition and cancer stem cells contribute to breast cancer heterogeneity. *Journal of Cellular Physiology*.

[2] Palazzo A. F., Lee ES., Non-coding R. N. A. (2015). What is functional and what is junk?. *Frontiers in Genetics*.

[3] Abdollahzadeh R., Daraei A., Mansoori Y., Sepahvand M., Amoli M. M., Tavakkoly-Bazzaz J. (2019). Competing endogenous RNA (ceRNA) cross talk and language in ceRNA regulatory networks: a new look at hallmarks of breast cancer. *Journal of Cellular Physiology*.

[4] Tuersong T., Li L., Abulaiti Z., Feng S. (2019). Comprehensive analysis of the aberrantly expressed lncRNA-associated ceRNA network in breast cancer. *Molecular Medicine Reports*.

[5] El-Ashmawy N. E., Hussien F. Z., El-Feky O. A., Hamouda S. M., Al-Ashmawy G. M. (2020). Serum LncRNA-ATB and FAM83H-AS1 as diagnostic/prognostic non-invasive biomarkers for breast cancer. *Life Sciences*.

[6] Mulrane L., McGee S. F., Gallagher W. M., O’Connor D. P. (2013). miRNA dysregulation in breast cancer. *Cancer Research*.

[7] Xia W., Liu Y., Cheng T., Xu T., Dong M., Hu X. (2020). Down-regulated lncRNA SBF2-AS1 inhibits tumorigenesis and progression of breast cancer by sponging microRNA-143 and repressing RRS1. *Journal of Experimental & Clinical Cancer Research*.

[8] Chen H., Shan G. (2020). Journal pre-proof the. Non-coding RNA research. *Noncoding RNA Research*.

[9] Brex D., Barbagallo C., Mirabella F. (2020). LINC00483 has a potential tumor-suppressor role in colorectal cancer through multiple molecular axes. *Frontiers in Oncology*.

[10] Liu H., Zhang Z., Wu N. (2018). Integrative analysis of dysregulated lncRNA-associated ceRNA network reveals functional lncRNAs in gastric cancer. *Genes*.

[11] Luo X., Tu T., Zhong Y. (2021). ceRNA network analysis shows that lncRNA CRNDE promotes progression of glioblastoma through sponge mir-9-5p. *Frontiers in Genetics*.

[12] Wong N. K., Huang C. L., Islam R., Yip S. P. (2018). Long non-coding RNAs in hematological malignancies: translating basic techniques into diagnostic and therapeutic strategies. *Journal of Hematology & Oncology*.

[13] Lemos E., Rodrigues N., Corre D. F., Dieter C. (2021). The impact of lncRNAs in diabetes mellitus: a systematic review and in. *Silico Analyses*.

[14] Rotondo J. C., Lanzillotti C., Mazziotta C., Tognon M., Martini F. (2021). Epigenetics of male infertility: the role of DNA methylation. *Frontiers in Cell and Developmental Biology*.

[15] Fang Y., Xu Y., Wang R. (2020). Recent advances on the roles of LncRNAs in cardiovascular disease. *Journal of Cellular and Molecular Medicine*.

[16] Zhang G., Pian C., Chen Z. (2018). Identification of cancer-related miRNA-lncRNA biomarkers using a basic miRNA-lncRNA network. *PLoS One*.

[17] Ko S., Kim J. Y., Jeong J., Lee J. E., Yang W. I., Jung W. H. (2014). The role and regulatory mechanism of 14-3-3 sigma in human breast cancer. *Journal of Breast Cancer*.

[18] Friess H., Ding J., Kleeff J. (2003). Microarray-based identification of differentially expressed growth- and metastasis-associated genes in pancreatic cancer. *Cellular and Molecular Life Sciences*.

[19] Villaret D. B., Wang T., Dillon D. (2000). Identification of genes overexpressed in head and neck squamous cell carcinoma using a combination of complementary DNA subtraction and microarray analysis. *Laryngoscope*.

[20] Perathoner A., Pirkebner D., Brandacher G. (2005). 14-3-3*σ* expression is an independent prognostic parameter for poor survival in colorectal carcinoma patients. *Clinical Cancer Research*.

[21] Kim Y., Shiba-Ishii A., Nakagawa T. (2018). Stratifin regulates stabilization of receptor tyrosine kinases via interaction with ubiquitin-specific protease 8 in lung adenocarcinoma. *Oncogene*.

[22] Sirivatanauksorn V., Dumronggittigule W., Dulnee B. (2020). Role of stratifin (14-3-3 sigma) in adenocarcinoma of gallbladder: a novel prognostic biomarker. *Surgical Oncology*.

[23] Young G. M., Radhakrishnan V. M., Centuori S. M., Gomes C. J., Martinez J. D. (2015). Comparative analysis of 14-3-3 isoform expression and epigenetic alterations in colorectal cancer. *BMC Cancer*.

[24] Kaneuchi M., Sasaki M., Tanaka Y. (2004). Expression and methylation status of 14-3-3 sigma gene can characterize the different histological features of ovarian cancer. *Biochemical and Biophysical Research Communications*.

[25] Moreira J. M. A., Ohlsson G., Rank F. E., Celis J. E. (2005). Down-regulation of the tumor suppressor protein 14-3-3*σ* is a sporadic event in cancer of the breast. *Molecular & Cellular Proteomics*.

[26] Moreira J. M. A., Gromov P., Celis J. E. (2004). Expression of the tumor suppressor protein 14-3-3*σ* is down-regulated in invasive transitional cell carcinomas of the urinary bladder undergoing epithelial-to-mesenchymal transition. *Molecular & Cellular Proteomics*.

[27] Liu Y., Chen Q., Zhang J.-T. (2004). Tumor suppressor gene 14-3-3*σ* is down-regulated whereas the proto-oncogene translation elongation factor 1*δ* is up-regulated in non-small cell lung cancers as identified by proteomic profiling. *Journal of Proteome Research*.

[28] Lodygin D., Hermeking H. (2005). The role of epigenetic inactivation of 14-3-3*σ* in human cancer. *Cell Research*.

[29] Liberzon A., Birger C., Thorvaldsdóttir H., Ghandi M., Mesirov J. P., Tamayo P. (2015). The molecular signatures database hallmark gene set collection. *Cell Systems*.

[30] Liberzon A., Subramanian A., Pinchback R., Thorvaldsdottir H., Tamayo P., Mesirov J. P. (2011). Molecular signatures database (MSigDB) 3.0. *Bioinformatics*.

[31] Subramanian A., Tamayo P., Mootha V. K. (2005). Gene set enrichment analysis: a knowledge-based approach for interpreting genome-wide expression profiles. *Proceedings of the National Academy of Sciences*.

[32] Ritchie M. E., Phipson B., Wu D. (2015). Limma powers differential expression analyses for RNA-sequencing and microarray studies. *Nucleic Acids Research*.

[33] Gautier L., Cope L., Bolstad B. M., Irizarry R. A. (2004). Affy–analysis of affymetrix genechip data at the probe level. *Bioinformatics*.

[34] Dweep H., Sticht C., Pandey P., Gretz N. (2011). MiRWalk—database: prediction of possible miRNA binding sites by “walking” the genes of three genomes. *Journal of Biomedical Informatics*.

[35] Fukunaga T., Iwakiri J., Ono Y., Hamada M. (2019). LncRRIsearch: a web server for lncRNA-RNA interaction prediction integrated with tissue-specific expression and subcellular localization data. *Frontiers in Genetics*.

[36] Paraskevopoulou M. D., Georgakilas G., Kostoulas N. (2013). DIANA-LncBase: experimentally verified and computationally predicted microRNA targets on long non-coding RNAs. *Nucleic Acids Research*.

[37] Jabbari N., Nawaz M., Rezaie J. (2019). Ionizing radiation increases the activity of exosomal secretory pathway in MCF-7 human breast cancer cells: a possible way to communicate resistance against radiotherapy. *International Journal of Molecular Sciences*.

[38] Sayar N., Karahan G., Konu O., Bozkurt B., Bozdogan O., Yulug I. G. (2015). Transgelin gene is frequently downregulated by promoter DNA hypermethylation in breast cancer. *Clinical Epigenetics*.

[39] Tang Z., Kang B., Li C., Chen T., Zhang Z. (2019). GEPIA2: an enhanced web server for large-scale expression profiling and interactive analysis. *Nucleic Acids Research*.

[40] Perkins J. R., Dawes J. M., Mcmahon S. B., Bennett D. L. H., Orengo C., Kohl M. (2012). ReadqPCR and NormqPCR: R packages for the reading, quality checking and normalisation of RT-qPCR quantification cycle (Cq) data. *BMC Genomics*.

[41] Jensen L. J., Kuhn M., Stark M. (2009). STRING 8–a global view on proteins and their functional interactions in 630 organisms. *Nucleic Acids Research*.

[42] Majeed W., Aslam B., Javed I. (2014). Breast cancer: major risk factors and recent developments in treatment. *Asian Pacific Journal of Cancer Prevention*.

[43] Müller S., Raulefs S., Bruns P. (2015). Next-generation sequencing reveals novel differentially regulated mRNAs, lncRNAs, miRNAs, sdRNAs and a piRNA in pancreatic cancer. *Molecular Cancer*.

[44] Zhang J., Liu L., Li J., Le T. D. (2018). LncmiRSRN: identification and analysis of long non-coding RNA related miRNA sponge regulatory network in human cancer. *Bioinformatics*.

[45] Raziq K., Cai M., Dong K., Wang P., Afrifa J., Fu S. (2020). Competitive endogenous network of lncRNA, miRNA, and mRNA in the chemoresistance of gastrointestinal tract adenocarcinomas. *Biomedicine & Pharmacotherapy*.

[46] Zhang Q., Liu H. M., Lv W. Q. (2018). Additional common variants associated with type 2 diabetes and coronary artery disease detected using a pleiotropic cFDR method. *Journal of Diabetes and its Complications*.

[47] Yu W., Xiang D., Jia H. (2020). The lncRNA BCYRN1 functions as an oncogene in human glioma by downregulating miR-125a-5p in vitro. *Cancer Management and Research*.

[48] Shiba-Ishii A., Noguchi M. (2012). Aberrant stratifin overexpression is regulated by tumor-associated CpG demethylation in lung adenocarcinoma. *American Journal Of Pathology*.

[49] Mhawech P., Benz A., Cerato C. (2005). Downregulation of 14-3-3*σ* in ovary, prostate and endometrial carcinomas is associated with CpG island methylation. *Modern Pathology*.

[50] Osada H., Tatematsu Y., Yatabe Y. (2002). Frequent and histological type-specific inactivation of 14-3-3*σ* in human lung cancers. *Oncogene*.

[51] Iwata N., Yamamoto H., Sasaki S. (2000). Frequent hypermethylation of CpG islands and loss of expression of the 14-3-3 *σ* gene in human hepatocellular carcinoma. *Oncogene*.

[52] Oh S., Shin S., Lightfoot S. A., Janknecht R. (2013). 14-3-3 proteins modulate the ETS transcription factor ETV1 in prostate cancer. *Cancer Research*.

[53] Meeran S. M., Patel S. N., Tollefsbol T. O. (2010). Sulforaphane causes epigenetic repression of hTERT expression in human breast cancer cell lines. *PLoS One*.

[54] Mühlmann G., Öfner D., Zitt M. (2010). 14-3-3 sigma and p53 expression in gastric cancer and its clinical applications. *Disease Markers*.

[55] Neupane D., Korc M. (2008). 14-3-3*σ* modulates pancreatic cancer cell survival and invasiveness. *Clinical Cancer Research*.

[56] Syren P., Andersson R., Bauden M., Ansari D. (2017). Epigenetic alterations as biomarkers in pancreatic ductal adenocarcinoma. *Scandinavian Journal of Gastroenterology*.

[57] Ren H.-Z., Pan G. q., Wang J.-S. (2010). Reduced stratifin expression can serve as an independent prognostic factor for poor survival in patients with esophageal squamous cell carcinoma. *Digestive Diseases and Sciences*.

[58] Dou M., Zhou X., Li L. (2021). Illumination of molecular pathways in multiple sclerosis lesions and the immune mechanism of matrine treatment in EAE, a mouse model of MS. *Frontiers in Immunology*.

[59] Sullivan K. D., Galbraith M. D., Andrysik Z., Espinosa J. M. (2018). Mechanisms of transcriptional regulation by p53. *Cell Death & Differentiation*.

[60] Yang H.-Y., Wen Y.-Y., Chen C.-H., Lozano G., Lee M.-H. (2003). 14-3-3*σ* positively regulates p53 and suppresses tumor growth. *Molecular and Cellular Biology*.

[61] Zhang Y., Karas M., Zhao H., Yakar S., LeRoith D. (2004). 14-3-3*σ* mediation of cell cycle progression is p53-independent in response to insulin-like growth factor-I receptor activation. *Journal of Biological Chemistry*.

[62] Wu Q., Fan H., Lang R. (2020). Overexpression of 14-3-3 *σ* modulates cholangiocarcinoma cell survival by PI3K/akt signaling. *BioMed Research International*.

[63] Du Z., Lovly C. M. (2018). Mechanisms of receptor tyrosine kinase activation in cancer. *Molecular Cancer*.

[64] Ogawara Y., Kishishita S., Obata T. (2002). Akt enhances mdm2-mediated ubiquitination and degradation of p53. *Journal of Biological Chemistry*.

[65] Guweidhi A., Kleeff J., Giese N. (2004). Enhanced expression of 14-3-3sigma in pancreatic cancer and its role in cell cycle regulation and apoptosis. *Carcinogenesis*.

